# A Novel Algorithm for the Evaluation of Corneal Nerve Beadings by *in vivo* Confocal Microscopy in Patients With Type 1 Diabetes Mellitus

**DOI:** 10.3389/fmed.2022.897259

**Published:** 2022-05-12

**Authors:** Irene Abicca, Daniela Giannini, Marta Gilardi, Anna Maria Roszkowska, Mariacristina Parravano, Fabiana Picconi, Simona Frontoni, Domenico Schiano-Lomoriello

**Affiliations:** ^1^IRCCS–Fondazione Bietti, Rome, Italy; ^2^Ophthalmic Hospital of Rome, Rome, Italy; ^3^Ophthalmology Clinic, Department of Biomedical Sciences, University of Messina, Messina, Italy; ^4^Unit of Endocrinology, Diabetes and Metabolism, S. Giovanni Calibita, Fatebenefratelli Hospital, Rome, Italy; ^5^Department of Systems Medicine, University of Rome Tor Vergata, Rome, Italy

**Keywords:** beading, corneal confocal microscopy, diabetes mellitus, corneal nerve, peripheral neuropathy

## Abstract

**Purpose:**

Peripheral neuropathy could complicate diabetes mellitus (DM). *In vivo* confocal microscopy (IVCM) is an ocular examination for the diagnosis of small fiber neuropathies and the detection of the earliest corneal sub-basal nerve plexus (SBP) alterations. Corneal SBP characteristics include focal enlargement along with the nerve fiber, called corneal beadings. These dilatations represent a mitochondrial accumulation induced by the reactive oxygen stress, as a consequence of hyperglycemia. For this reason, corneal beadings are considered indicative of metabolic activity. This study aimed to describe the corneal characteristics of a population of type 1 diabetes mellitus (T1DM) well metabolically controlled, using a new algorithm for the analysis of corneal beading size (BS).

**Methods:**

Patients aged ≥18 years affected by T1DM were compared with healthy subjects who underwent IVCM (Confoscan 4; Nidek Technologies Padova, Italy). Starting from the coordinates of the beadings detected by the IVCM, we implemented a new algorithm for automatically measuring BS in corneal SBP images.

**Results:**

We compared 20 eyes of T1DM patients with 26 healthy controls. The corneal nerves' fiber length (*p* = 0.008), corneal nerves' fiber length density (*p* = 0.008), and the number of fibers (*p* = 0.017) were significantly lower in the diabetic group compared with controls. There was no difference between diabetic and healthy eyes in the mean number of corneal beadings both in the frame of analysis (*p* = 0.606) and for 0.1 mm of SBP nerve (*p* = 0.145). Regarding the BS, patients with T1DM had corneal beadings larger than controls (*p* = 0.036).

**Conclusions:**

We found that the corneal beadings parameters are similar in healthy and T1DM individuals. Nevertheless, measuring the BS with our algorithm, we showed that corneal beadings are enlarged in patients affected by T1DM when compared with healthy controls. Identifying beading expansion in corneal nerve fiber using IVCM should become a useful tool to predict peripheral neuropathy at an early stage.

## Introduction

Type 1 diabetes mellitus (T1DM) is one of the most common metabolic disorders worldwide. A severe complication, which causes disability and reduces the quality of life (1), is represented by diabetic peripheral neuropathy (DPN), which is frequently diagnosed at a late stage. DPN should include corneal sensitivity alteration, with corneal sub-basal nerve plexus (SBP) modifications (2, 3). Furthermore, SBP damages have been described to be related to the duration of the disease and unsuitable glycemic control (4, 5).

Skin biopsy is considered the gold standard for the evaluation of morphological change in small nerve fibers and diabetic neuropathy progression (6). Unfortunately, this procedure is characterized by invasive nature and increased costs and can lead to delayed healing of the bioptic area (7). Therefore, it cannot be used as a screening test for all patients with diabetes. Alternatively, *in vivo* confocal microscopy (IVCM) is a rapid and reproducible ophthalmic application that allows studying of all corneal layers, such as cellular elements and small nerve fibers in the corneal SBP (2, 8, 9).

It has been proven that intraepidermal nerve fiber density and corneal nerve fiber morphology, evaluated by IVCM, are both correlated to neuropathy stages (10). Due to this, IVCM is considered an efficient non-invasive, and reliable alternative to skin biopsy, able to diagnose and track the progression of peripheral neuropathy in patients with diabetes (11).

In particular, the SBP analysis carried out by IVCM revealed a significant reduction in corneal parameters, specifically for nerve fibers length, nerve fibers length density, number of fibers, bifurcations, and beadings in patients with diabetes (12). Glycosylated hemoglobin levels in patients with diabetes showed an inverse relationship with nerve fibers length, nerve fibers length density, branches, and number of beadings (12).

The pathogenetic mechanism of the onset of diabetic neuropathy seems to be the reactive oxygen stress induced by hyperglycemia.

One of the consequences of reactive oxygen stress is to induce the accumulation of mitochondria along with the nerves fibers (13). In particular, beadings are enlargements (beads) along nerve fibers, which appear hyper-reflective at the IVCM examination. These dilatations are considered indicative of metabolic activity (14, 15).

Several studies reported the corneal nerve fibers changes in patients with diabetes (16–19), compared with healthy controls and described the beadings characteristics as number and density. Some of these studies suggested a direct correlation between the decrease of beading frequency (17–19) and metabolic activity of nerve fibers in patients with diabetes. Furthermore, the beading size (BS) resulted in being altered in diabetes, as described by Ishibashi et al. (17), using their specific algorithm. They reported a beading frequency reduction associated with a bead enlargement in a patient with type 2 diabetes, compared with healthy subjects. In addition, the number and size of beads were found to be related to changes in the mitochondrial distribution along corneal nerve fibers. Altered beading structures may have a relationship with peripheral nerve changes, therefore, the BS might be a new biomarker for the detection of DPN at its earliest stage.

Nowadays, despite the evidence in the literature regarding the potential role of the beading dimension in predicting diabetic neuropathy evolution, a reproducible method for evaluating this characteristic is not in use.

This study aimed to apply a new algorithm for the measurement of corneal BS in the corneas of a T1DM population and compare it with a healthy control group.

## Materials and Methods

### Study Population

We enrolled patients aged ≥18 years affected by T1DM according to the American Diabetes Association (ADA) criteria (20), referring to the Unit of Endocrinology, Diabetes, and Metabolism, Department of Systems Medicine in S. Giovanni Calibita Fatebenefratelli Hospital, University of Rome Tor Vergata, Rome, Italy, and we compared them with a control group of healthy adults, matched by age. All patients underwent a general medical examination, anthropometric parameters, and laboratory measurements. Blood and urinary samples were analyzed as previously described by our group (16). Triglycerides (TG), plasma total cholesterol (TC), high and low-density lipoprotein cholesterol (HDL-C and LDL-C), glycosylated hemoglobin (HbA1c), creatinine and microalbuminuria (urinary albumin/creatinine ratio) were performed in all T1DM, to rule out the confounding effect of high lipid values or renal failure on corneal nerves parameters.

We excluded diabetic patients with microalbuminuria >30 mg/g and diabetic autonomic neuropathy (DAN) evaluated by Ewing battery (21).

Regarding the healthy subjects, we performed an oral glucose tolerance test to exclude diabetes and impaired glucose tolerance. In addition, we excluded subjects with dyslipidemia, chronic renal failure, and hypertension based on their medical history.

In both groups (T1DM and control), we included patients with no history of possible confounding diseases (inflammatory diseases, alcohol abuse, vitamin deficiency, malignancy treated with chemotherapy agents, recent history of heart or respiratory failure, chronic liver or renal failure central nervous system diseases, entrapment mononeuropathies, and cervical or lumbosacral radiculopathies). Ocular exclusion criteria in all subjects were contact lens wearing, ocular trauma, ocular medications (except for artificial tears), clinical history of eye surgery, and ocular inflammation.

### *In vivo* Confocal Microscopy

*In vivo* confocal microscopy (Confoscan 4; Nidek Technologies Padova, Italy) was performed bilaterally on the central cornea of all patients that included with a Z-ring adapter.

The same experienced operator (DSL) achieved all examinations. We used a sterile and transparent gel (dexpanthenol 5%) on the top of the instrumental lens to eliminate optical interfaces and to keep no-contact examination with invasiveness. After auto-alignment, a full-thickness scan of the cornea was performed, as previously described (22). The overall time for the examination was up to 3 min. Some patients complained of ocular symptoms or visual complications.

Two experienced researchers (IA and MG), masked by the group assignment of images, carefully examined the images. One eye for each patient was considered, choosing the best-focused frame of the SBP for each patient. They discarded images with motions and/or with more than one layer.

The CS4 software (Nerves Tracking Tool v1.3.0) was used to automatically identify corneal fibers and beadings in each frame and review them. Errors were manually edited. In the case of a mismatch, a third operator (DG) chose the best option.

The following SBP parameters were available by the instrument for the analysis:

1. Nerve fiber length (μm/frame): the total length of all fibers and branches in a frame;
2. Nerve fiber length density (μm/mm^2^): the total density of the nerve fibers in mm^2^;
3. Number of fiber: the total number of nerve fibers, including main nerves and branches;
4. Number of branching: points where nerve branches arise from the main nerve;
5. Nerve fiber tortuosity using Nidek Nerve index, a unitless measure that represents the degree of the twistedness of a curved structure;
6. Number of beadings: the total number of beadings identified in the main nerves (trunks, long fibers that crossed the borders of the area of analysis);
7. Beadings density (beadings/mm): the total number of beadings divided by the total length of nerve trunks in millimeters.

**Figure 1:**
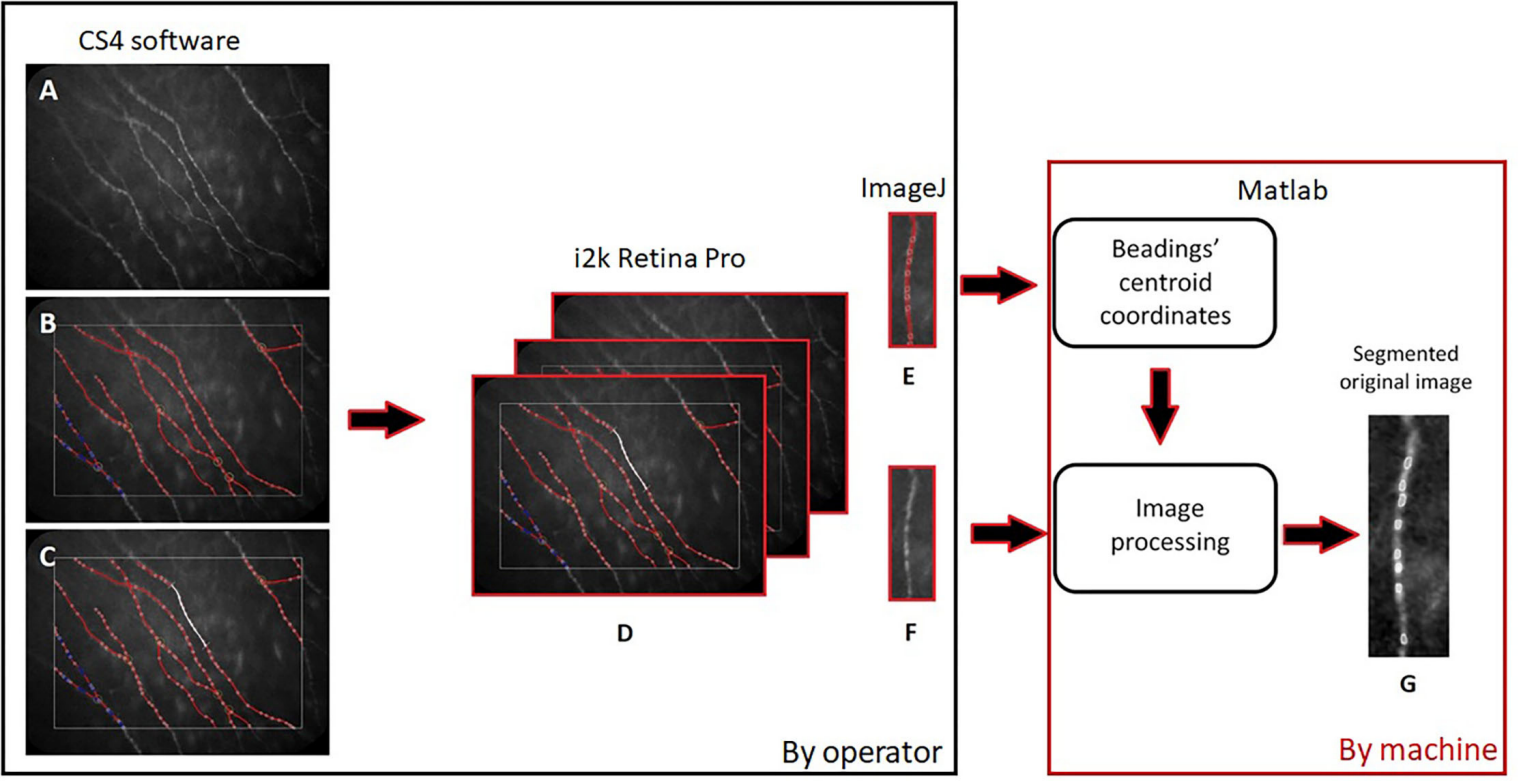
Diagram of images processing: The original image of nerves **(A)**, the one with the overlaying analysis and beadings identification **(B)**, and the capture with 0.1 mm nerve portion **(C)** were aligned **(D)**, vertically rotated **(E,F)** by the operator (black frame on the left) and finally processed using our automatic algorithm **(G)** (red frame on the right).

After the SBP detection using CS4 software, as described above, we processed the images to obtain a new morphological characterization of the beadings. To achieve this we saved three different images of the same capture of fibers:

1. The original image of nerves (Figure 1A);
2. The image with the overlaying analysis of the nerves and beadings identification (Figure 1B);
3. The capture with a manual selection of 0.1 mm nerve portion using the caliper tool (Figure 1C).

**Figure 2:**
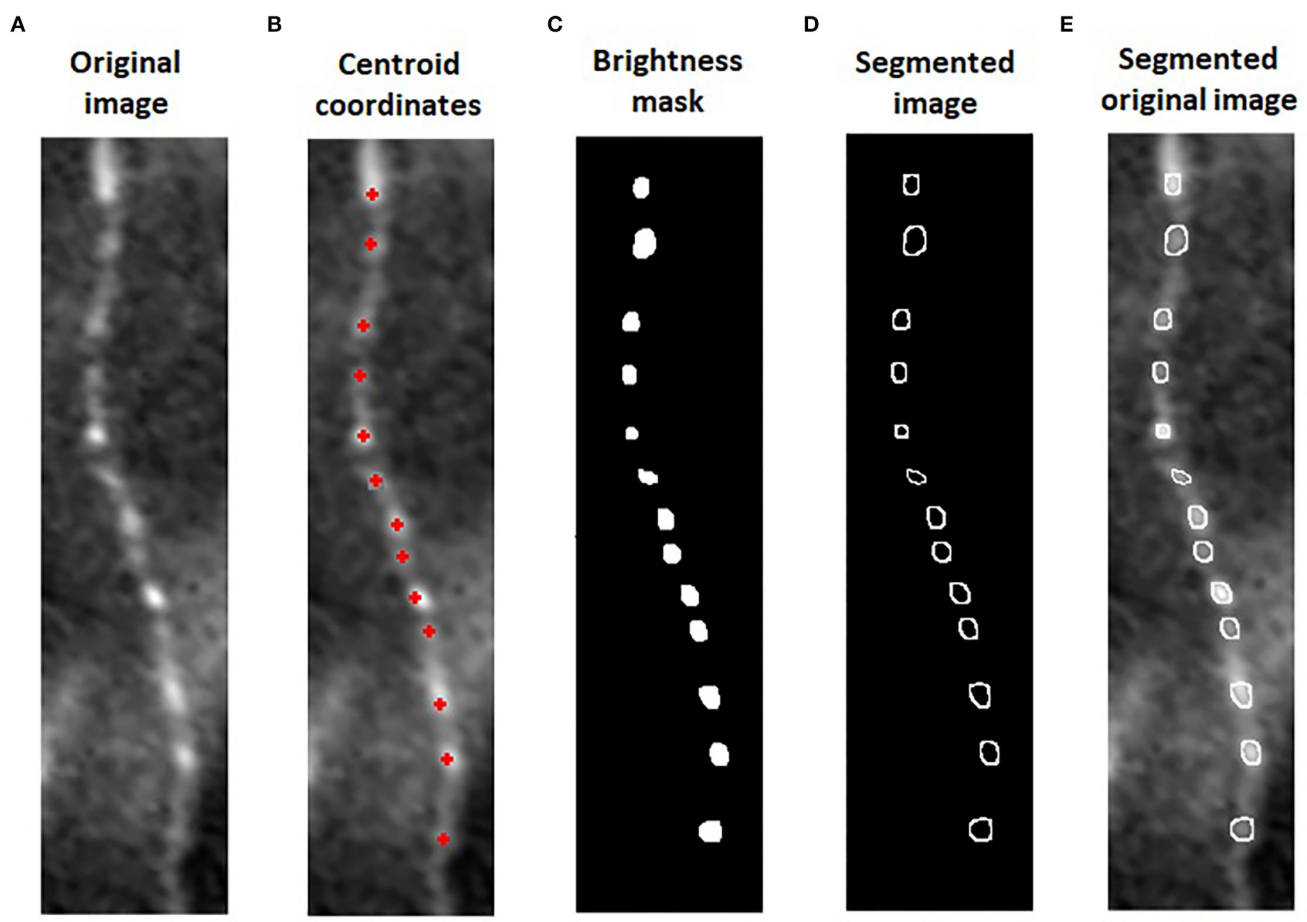
Algorithm for automatic beading size (BS) measure: We cropped 0.1 mm of nerve in the original image of sub-basal plexus **(A)**. The algorithm used the coordinates of corneal beadings' centroids from the image with overlaying analysis **(B)** to segment the area and defined the beadings in the original image. The intensity of each centroid was used to mask all the pixels below this value **(C)**. On the original image, the perimeter of the beadings was segmented and the BS quantified **(D,E)**.

We aligned these three images (Figure 1D), using i2k Retina Pro (version 3.1), and we vertically rotated the fiber and cropped 0.1 mm of corneal nerve, using ImageJ (Figures 1E,F). After this preparation of images, a customized algorithm was applied to automatically measure BS in corneal nerves (Figure 1G). The algorithm was developed using Matlab (version R2015a; The Mathworks, Inc., Natick, MA, USA). Previously, the operator, using *imtool* (Image Processing Toolbox Matlab), extracted the coordinates of corneal beadings' centroids (defined as the geometric center of a plane figure) from the image with overlaying beadings analysis. The algorithm used these coordinates from the image with overlaying analysis to automatically segment the area and defined the BS in the original image (Figures 2A,B).

On one beading at a time, the intensity of each centroid was used by the algorithm as a threshold to filter all the pixels with a brightness below this value. Finally, a mask overlap was created on the original image to automatically border the perimeter of the beadings (Figures 2C,D). The beading area was quantified from this segmentation (Figure 2E). For each patient, we calculated the BS, defined as the sum of all areas divided by the number of corneal beadings identified on 0.1 mm of the nerve fiber.

### Statistical Analysis

The SPSS (IBM SPSS Statistics 25) was used for statistical evaluation. All results were expressed as the mean ± standard deviations (SDs). The independent sample *t*-test or the Mann–Whitney test was used for statistical analysis, as appropriate. In all analyses, *p* < 0.05 was considered to be statistically significant.

## Results

We included twenty eyes of 20 patients affected by T1DM, and we compared them to 26 healthy controls. The groups were similar in age (*p* = 0.583). The clinical and demographic characteristics of the study population are described in Table 1.

**Table 1:** Demographic and metabolic characteristics of study population (SD, standard deviation; T1DM, type 1 diabetes mellitus; DM, diabetes mellitus; BMI, body mass index; HBA1c, glycosylated hemoglobin; TC, total cholesterol; HDL-C, high-density lipoprotein cholesterol; LDL-C, low-density lipoprotein; TG, triglycerides).

	***Healthy*** ***(n = 26)***	***T1DM*** ***(n = 20)***
Age (mean ± SD)	42.73 ± 12.18	40.50 ± 15.15
Sex (male/female)	13/13	9/11
Age at diagnosis of DM (y)	–	25.15 ± 14.20
Duration of DM (y)	–	15.35 ± 12.66
BMI (Kg/m^2^)	–	23.39 ± 3.10
HBA1c (%)	–	7.74 ± 1.00
TC (mg/dl)	–	165.60 ± 34.26
HDL-C (mg/dl)	–	58.75 ± 13.22
LDL-C (mg/dl)	–	91.46 ± 26.78
TG (mg/dl)	–	76.50 ± 38.18
Creatinine (mg/dl)	–	0.78 ± 0.11
Microalbuminuria/creatininuria (mg/g)	–	7.93 ± 7.11

All patients underwent IVCM and, using CS4 software (CS4 Nerves Tracking Tool v1.3.0), we analyzed their SBP. The corneal nerves' fiber length, the length density, and the number of fibers were lower in the diabetic group and these differences were statistically significant (Table 2).

**Table 2:** Sub-basal plexus corneal nerves parameters of study population (SD, standard deviation; T1DM, type 1 diabetes mellitus; *statistically significant).

***Nerve parameters*** ***(mean* ± SD)**	***Healthy*** ***(n = 26)***	***T1DM*** ***(n = 20)***	***P-value***
Nerve fibers length (μm/frame)	1216.03 ± 378.05	965.40 ± 233.52	0.008*
Nerve fibers length density (μm /mm^2^)	13690.3 ± 4246.7	10863.24 ± 2627.64	0.008*
Number of fibers (n°/mm^2^)	6.65 ± 2.56	5.15 ± 1.53	0.017*
Number of branching	2.69 ± 1.83	1.75 ± 1.25	0.085
Nerve nerve fiber tortuosity	5.48 ± 1.61	5.31 ± 1.33	0.973
Beadings density (n°/mm)	68.92 ± 13.96	63.87 ± 14.75	0.278
Beadings number (n°/frame)	20.65 ± 11.46	16.85 ± 3.91	0.606
Beading size (μm^2^)	11.11 ± 2.5	12.94 ± 3.05	0.036*

There was no difference between diabetic and healthy eyes in a mean number of corneal beadings in the frame analyzed (*p* = 0.606).

We cropped 0.1 mm of SBP nerve for the analysis in our new algorithm. Using the corneal beadings' detection of CS4 software with the manual correction (as described in the Materials and methods Section), we found that, even in this case, the mean number of corneal beadings was similar between the two groups (T1DM = 7.8 ± 2.17 vs. control = 8.85 ± 1.69, *p* = 0.145).

Then we applied our new algorithm to all images for the automatic measurement of BS both in T1DM and in the control group. We found that, in patients with T1DM, BS was higher than in healthy controls and this difference was statistically significant (T1DM = 12.94 ± 3.05 μm^2^ vs. control = 11.11 ± 2.5 μm^2^, *p* = 0.031).

Comparing T1DM without and with DPN (12 eyes vs. 8 eyes, respectively), we found that they were similar in age and metabolic characteristics. Furthermore, we did not find any difference in corneal parameters between T1DM and BS (Table 3).

**Table 3:** Comparison of sub-basal plexus demographic, metabolic and corneal nerves parameter of T1DM with and without diabetic peripheral neuropathy (DPN) (SD, standard deviation; T1DM, type 1 diabetes mellitus; DM, diabetes mellitus; BMI, body mass index; HBA1c, glycosylated hemoglobin; TC, total cholesterol; HDL-C, high-density lipoprotein cholesterol; LDL-C, low-density lipoprotein; TG, triglycerides).

***Nerve parameters***	***T1DM without DPN*** ***(n = 12)***	***T1DM with DPN*** ***(n = 8)***	***P-value***
Age (mean ± SD)	41.08 ± 15.22	39.63 ± 16.06	0.842
Sex (male/female)	2/10	6/2	
Age at onset of DM (y)	30.08 ± 15.99	17.75 ± 6.41	0.157
Duration of DM (y)	11.00 ± 6.31	21.88 ± 17.07	0.181
BMI (Kg/m^2^)	23.05 ± 2.06	23.91 ± 4.34	0.615
HBA1c (%)	7.53 ± 0.89	8.06 ± 1.12	0.277
TC (mg/dl)	169.83 ± 35.41	159.25 ± 33.74	0.678
HDL-C (mg/dl)	64.67 ± 13.01	49.88 ± 7.64	0.005
LDL-C (mg/dl)	90.92 ± 28.96	92.28 ± 25.04	0.624
TG (mg/dl)	70.50 ± 32.5	85.50 ± 46.28	0.384
Creatinine (mg/dl)	0.75 ± 0.11	0.83 ± 0.09	0.135
Microalbuminuria/ creatininuria (mg/g)	9.34 ± 7.81	5.82 ± 5.71	0.260
Nerve fibers length (μm/frame)	957.65 ± 257.42	977.03 ± 208.86	0.855
Nerve fibers length density (μm /mm^2^)	10776.04 ± 2896.63	10994.04 ± 2350.15	0.855
Number of fibers (n°/mm^2^)	5.5 ± 1.68	4.63 ± 1.19	0.189
Number of branching	2.08 ± 1.51	1.25 ± 0.46	0.270
Nerve fiber tortuosity	5.23 ± 1.52	5.43 ± 1.08	0.735
Beadings density (n°/mm)	64.55 ± 18.22	62.86 ± 8.16	0.270
Beadings number (n°/frame)	16.17 ± 4.59	17.88 ± 2.53	0.300
Beading size (μm^2^)	13.25 ± 3.50	12.47 ± 2.36	0.560

## Discussion

Peripheral neuropathy is a severe complication of both type 1 and type 2 diabetes mellitus (T1DM and T2DM). The recommendations of the American Diabetes Association are to assess annually for DPN in all patients with T2DM and patients with a diagnosis of T1DM for more than 5 years (23).

*In vivo* confocal microscopy is a reliable, non-invasive method for the detection of DPN, including small nerve fiber lesions, even in patients without symptoms (10, 24).

The cornea is one of the most richly innervated body tissues, with a sensitive innervation provided by the ophthalmic branch of the trigeminal nerve. The corneal nerves allow for protecting, restoring, and supporting the ocular surface (25). Consequently, IVCM, which enables the *in vivo* analysis of corneal innervation, is a useful tool both for the study of the peripheral nerves involvement and for the identification of risk factors for ocular surface disorders in patients with diabetes.

Hyperglycemia induces reactive oxygen stress, playing an important role in diabetic neuropathy onset and leading to the accumulation of mitochondria along with the nerves fiber. At IVCM, mitochondria are visualized as corneal beadings. In T1DM, when the increase in superoxide induces the activation of the polyol pathway and the accumulation of glycosylation products, damage of ocular surface and corneal nerves occurs (26). The long-term effects of the metabolic and vascular disorders in diabetes are neural function impairment and loss of neurotrophic support, which induces apoptosis of neurons, Schwann cells, and glial cells of the peripheral nervous system, resulting in corneal nerves reduction.

Our group already reported early alterations in SBP even in a small population of T1DM adults without DPN and diabetic retinopathy (16). However, we did not find any difference in the number and density of beadings compared with healthy controls. According to these results, beadings should be excluded as a parameter for the early detection of corneal nerve alterations in T1DM patients with good metabolic control. Nevertheless, we inquired about another beading parameter that should be speculated as well: the size. For this reason, the purpose of the present study was to investigate the beading size in a population of T1DM. We chose patients who had good glycemic and lipid control because we aimed to eliminate confounding factors of the metabolism.

First, in our study, we analyzed the corneal nerve parameters at IVCM and we found that corneal fiber length, length density, and number of fibers were significantly lower in our patients with T1DM compared with controls. These results confirm that corneal nerves appeared altered even in a well metabolically controlled population of T1DM, as already reported by our group and in literature (16, 27).

As far as corneal beadings are concerned, we did not find differences in the number and density of corneal beadings between T1DM and controls.

According to the literature, patients affected by T1DM showed a lower beading frequency when compared with healthy controls (5, 17) and this seemed to be in contrast with our results. However, different methods for the analysis were applied. The “beading frequency” was defined as frequency/0.1 mm of beading, using ImageJ (Texelcraft, Tokyo, Japan), while we described in our studies the “beading density,” defined as the total number of beadings divided by the total length of nerve trunks in millimeters, using the automatic count of CS4 software (12) and manually corrected.

In our opinion, the lack of alteration in beading number and density should be the consequence of the good glycemic and lipid control of our diabetic population. However, we applied our new algorithm to speculate the size of beadings. We found that corneal beadings were bigger in patients affected by T1DM than in healthy controls and this difference was statistically significant.

Ishibashi et al. (17) already described the BS in a large cohort study of T2DM. They found that, even in absence of DPN, the worsening of peripheral neuropathy follows an enlargement of bead size.

To our knowledge, our study is the first to describe the enlargement of the new parameter BS in a T1DM population well metabolically controlled and without number and density alteration.

Hishibashi's group (5) already hypothesized that beadings alterations could be the consequence of changes in the distribution of mitochondria during diabetes mellitus.

Mitochondrial loss in the early stages of small fiber neuropathies was reported (28, 29).

The new and interesting result of our study was that, even in a group of patients with diabetes with a similar number and density of beadings compared with healthy controls, we had an alteration in bead size. This finding should be the consequence of mitochondrial dysfunction. The advantage of our new algorithm is the completely automatic measurement of BS, based on the previous analysis of beadings using the reproducible method of CS4 software (12).

The main limitation of our research was the small number of patients included, due to the strict inclusion criteria for the analysis. Therefore, a longitudinal study with a larger group should be further investigated.

Our goal for future developments is the application of our algorithm for the bead detection, making the analysis of the new BS parameter easier. Now, the limits of this analysis of corneal nerves are that a lot of time and specifically trained operators are needed. Furthermore, the study of corneal innervation allows us to speculate on the pathogenic mechanism of ocular surface alteration in diabetes. Due to the report on the impact of age on density and morphology of SBP in healthy adults (30), it should be interesting, in future studies, to evaluate the correlation between the age of patients with diabetes and the progression of corneal damage. The deepening of the analysis of the nerves and specifically of the corneal beadings, which are associated with metabolic activity in diabetes, could help to better understand these alterations.

In our opinion, identifying beading expansion in corneal nerve fiber using IVCM should become a useful tool for predicting peripheral neuropathy at an early stage.

## Data Availability Statement

The original data used and analyzed to support the findings of this study are included in the Supplementary Material, further inquiries can be directed to the corresponding author/s.

## Ethics Statement

The studies involving human participants were reviewed and approved by Sezione IRCCS I.F.O-Fondazione G.B. Bietti. The patients/participants provided their written informed consent to participate in this study.

## Author Contributions

IA, DG, and DSL: conception and design. IA, MP, FP, and SF: collection and assembly of data. IA, DG, MG, and AMR: data analysis and interpretation. All authors wrote the manuscript and final approval of the manuscript.

## Conflict of Interest

The authors declare that the research was conducted in the absence of any commercial or financial relationships that could be construed as a potential conflict of interest.

## Publisher's Note

All claims expressed in this article are solely those of the authors and do not necessarily represent those of their affiliated organizations, or those of the publisher, the editors and the reviewers. Any product that may be evaluated in this article, or claim that may be made by its manufacturer, is not guaranteed or endorsed by the publisher.
